# Supplementary figures and images for: Spatial population genetic structure of Caquetaia kraussii (Steindachner, 1878) evidenced by species-specific microsatellite loci in the middle and low basin of the Cauca River, Colombia
Source: PLoS One. 2024 Jun 4;19(6):e0304799. doi: 10.1371/journal.pone.0304799 (PMC11149877; doi:10.1371/journal.pone.0304799)

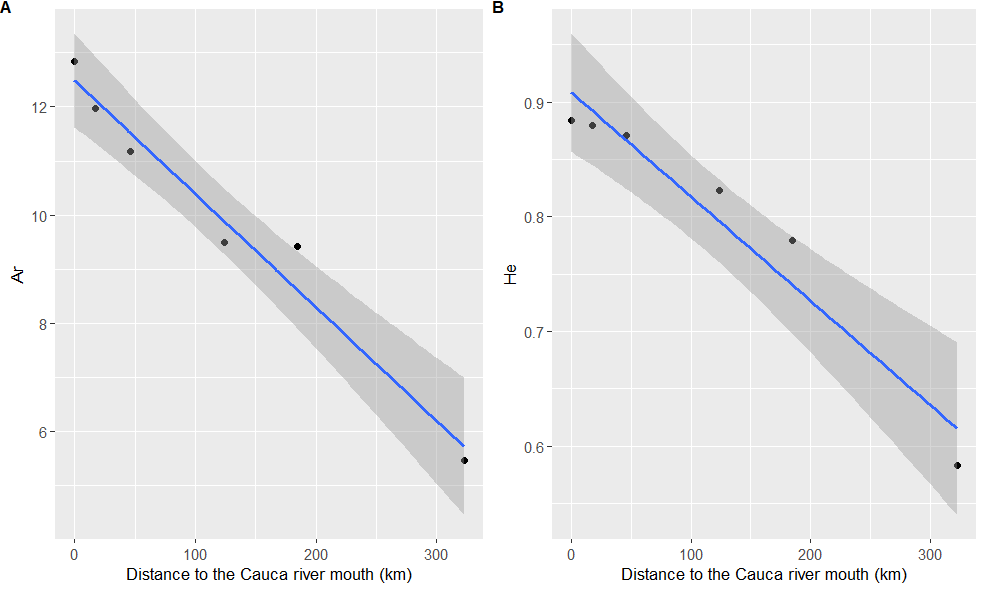

Supplement: S1 Fig — Ar: allelic richness; He: expected heterozygosity. He vs Distance to the Cauca River mouth (R: -0.970, p: 0.001). Ar vs Distance to the Cauca River mouth (R: 1.000, p < 0.0001). (TIF) [file pone.0304799.s001.tif]

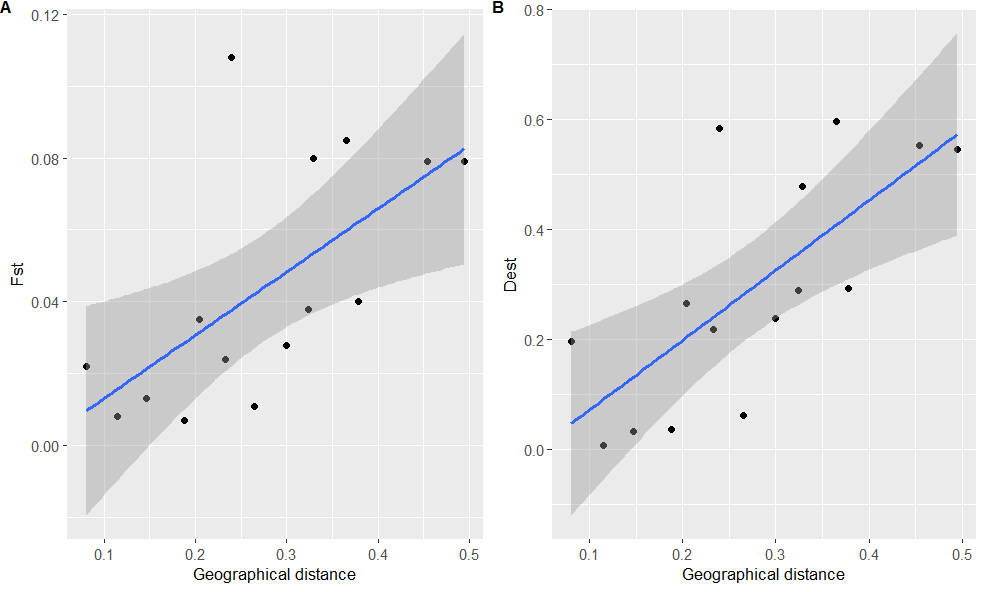

Supplement: S2 Fig — F’st vs Geographical distance: R = 0.633, p = 0.045. Jost’s DEST vs Geographical distance: R = 0.717, p = 0.009. (TIF) [file pone.0304799.s002.tif]
