# Supplementary material for: Spatial population genetic structure of Caquetaia kraussii (Steindachner, 1878) evidenced by species-specific microsatellite loci in the middle and low basin of the Cauca River, Colombia
Source: PLoS One. 2024 Jun 4;19(6):e0304799. doi: 10.1371/journal.pone.0304799 (PMC11149877; doi:10.1371/journal.pone.0304799)
Supplement: S1 Table — Ra: Allelic range. Na: number of alleles per locus. PIC: polymorphism information content. Tail A: GCCTCCCTCGCGCCA. Tail B: GCCTTGCCAGCCCGC. Tail C: CAGGACCAGGCTACCGTG. Tail D: CGGAGAGCCGAGAGGTG. (DOCX) [file pone.0304799.s003.docx]

| **Locus** | **Motif** | **Primer F (forward)** | **Primer R (reverse)** | **Ra** | **Na** | **PIC** | **Ho** | **He** | **P** | **Fis** | **Tail** | **Exclusion criteria** |
| --- | --- | --- | --- | --- | --- | --- | --- | --- | --- | --- | --- | --- |
| Ckra09 | ATGG | CCAGAACAAAATGCTCACTGC | AACCTGTCCAAGGTGTGCC | 157-185 | 6 | 0.619 | 0.474 | 0.688 | **0.041** | 0.318 | A | Inconsistent amplification |
| Ckra10 | ATTGG | CAATAGCCTACACTCTGGACAGG | TCATGAGGAACAGGAAAACAGG | 260 | 0 | 0.000 | 0.000 | 0.000 | **0.001** | 0.000 | A | Monomorphic |
| Ckra11 | ATGG | GGATGCTCATATTGAGCGTAACC | TCAGAGCCAGAAGGTGAGAGC | 285-309 | 6 | 0.561 | 0.632 | 0.620 | 0.210 | -0.019 | A | Inconsistent amplification |
| Ckra14 | ATGG | TCGCTTCATAGAAATGTTGTTGG | CATAATGAGTCGAACCAGGGC | 295-311 | 4 | 0.539 | 0.526 | 0.596 | 0.457 | 0.120 | A | Inconsistent amplification |
| Ckra15 | ATGG | ACACATGTCAGGTGGATGGG | ATTGTATCCTGCCTCTCGGC | 104-124 | 4 | 0.495 | 0.421 | 0.558 | 0.204 | 0.250 | A | Low polymorphism |
| Ckra16 | ATGG | AACATCCTGCAGCATTCACG | CTGTGTGCTTCCATCCATGC | 297-341 | 8 | 0.569 | 0.421 | 0.613 | **0.005** | 0.319 | A | Inconsistent amplification |
| Ckra17 | ATGG | ATGATGTGCTGATGGATGGG | GAGCTGGTTCGGGATGTAGG | 175-191 | 5 | 0.560 | 0.632 | 0.649 | 0.971 | 0.027 | D | Inconsistent amplification |
| Ckra19 | ATGG | AGACCCTGAACAGGATAAGTGG | TGAAACTTGCTGATCACAGCC | 154-206 | 11 | 0.778 | 0.474 | 0.824 | **0.001** | 0.432 | D | Inconsistent amplification |
| Ckra20 | ATGG | TGAAACAAACTGGTTGGAAGG | GGGCAGCATCAACTACAATGG | 239-259 | 6 | 0.313 | 0.211 | 0.334 | 0.056 | 0.377 | D | Low polymorphism |
| Ckra23 | ATGG | GTGGAGACGACACCAAGTGC | TGCATTAAGCAGCTGTTGCC | 172-216 | 9 | 0.497 | 0.474 | 0.528 | 0.356 | 0.105 | D | Low polymorphism |
| Ckra25 | ATGG | ACATGGAGCTGATTCCAGCC | AAAATGCAGTGGCAAAGAGC | 111-135 | 5 | 0.386 | 0.474 | 0.414 | 1.000 | -0.149 | C | Low polymorphism |
| Ckra26 | ATGG | CACCCTGTTGTGGTTAACGG | GCTGGTTCAATATCCGTCCG | 124-136 | 4 | 0.435 | 0.316 | 0.542 | 0.073 | 0.424 | C | Low polymorphism |
| Ckra28 | ATCT | CTGTGGCAGCTGGGATAAGC | ATCCCCAGAAAATGTGCAGC | 219-287 | 11 | 0.627 | 0.526 | 0.660 | **0.012** | 0.207 | C | Inconsistent amplification |
| Ckra30 | AGCC | TCCAAACACGGTCAGTCTGC | TGCTGCAACATTCCAGTGC | 241-245 | 2 | 0.313 | 0.316 | 0.398 | 0.549 | 0.212 | C | Low polymorphism |
