# Supplementary material for: Spatial population genetic structure of Caquetaia kraussii (Steindachner, 1878) evidenced by species-specific microsatellite loci in the middle and low basin of the Cauca River, Colombia
Source: PLoS One. 2024 Jun 4;19(6):e0304799. doi: 10.1371/journal.pone.0304799 (PMC11149877; doi:10.1371/journal.pone.0304799)
Supplement: S3 Table — (DOCX) [file pone.0304799.s005.docx]

| locus1 | locus2 | P value | locus1 | locus2 | P value | locus1 | locus2 | P value | locus1 | locus2 | P value |
| --- | --- | --- | --- | --- | --- | --- | --- | --- | --- | --- | --- |
| Ckra07 | Ckra22 | 0.003 | Ckra08 | Ckra27 | 0.172 | Ckra01 | Ckra04 | 0.454 | Ckra03 | Ckra24 | 0.727 |
| Ckra06 | Ckra24 | 0.004 | Ckra02 | Ckra21 | 0.178 | Ckra04 | Ckra27 | 0.454 | Ckra01 | Ckra27 | 0.732 |
| Ckra21 | Ckra22 | 0.012 | Ckra04 | Ckra24 | 0.184 | Ckra01 | Ckra03 | 0.464 | Ckra03 | Ckra21 | 0.735 |
| Ckra01 | Ckra06 | 0.014 | Ckra18 | Ckra22 | 0.189 | Ckra12 | Ckra27 | 0.470 | Ckra02 | Ckra24 | 0.745 |
| Ckra04 | Ckra13 | 0.017 | Ckra05 | Ckra07 | 0.195 | Ckra21 | Ckra24 | 0.474 | Ckra04 | Ckra06 | 0.748 |
| Ckra02 | Ckra22 | 0.017 | Ckra04 | Ckra12 | 0.202 | Ckra06 | Ckra27 | 0.490 | Ckra05 | Ckra06 | 0.748 |
| Ckra18 | Ckra29 | 0.018 | Ckra13 | Ckra24 | 0.211 | Ckra03 | Ckra13 | 0.492 | Ckra06 | Ckra22 | 0.754 |
| Ckra01 | Ckra07 | 0.030 | Ckra22 | Ckra27 | 0.242 | Ckra02 | Ckra04 | 0.508 | Ckra02 | Ckra05 | 0.784 |
| Ckra01 | Ckra02 | 0.036 | Ckra21 | Ckra27 | 0.248 | Ckra07 | Ckra13 | 0.528 | Ckra04 | Ckra07 | 0.801 |
| Ckra05 | Ckra12 | 0.040 | Ckra27 | Ckra29 | 0.253 | Ckra07 | Ckra12 | 0.531 | Ckra02 | Ckra06 | 0.809 |
| Ckra04 | Ckra05 | 0.055 | Ckra03 | Ckra29 | 0.254 | Ckra03 | Ckra04 | 0.544 | Ckra02 | Ckra27 | 0.836 |
| Ckra04 | Ckra22 | 0.060 | Ckra02 | Ckra13 | 0.271 | Ckra07 | Ckra29 | 0.556 | Ckra07 | Ckra08 | 0.849 |
| Ckra01 | Ckra21 | 0.062 | Ckra07 | Ckra18 | 0.272 | Ckra08 | Ckra22 | 0.556 | Ckra02 | Ckra12 | 0.854 |
| Ckra05 | Ckra24 | 0.074 | Ckra02 | Ckra18 | 0.290 | Ckra18 | Ckra21 | 0.559 | Ckra07 | Ckra24 | 0.856 |
| Ckra13 | Ckra18 | 0.079 | Ckra06 | Ckra07 | 0.294 | Ckra18 | Ckra24 | 0.562 | Ckra06 | Ckra08 | 0.859 |
| Ckra01 | Ckra29 | 0.090 | Ckra08 | Ckra18 | 0.296 | Ckra02 | Ckra08 | 0.567 | Ckra05 | Ckra22 | 0.859 |
| Ckra05 | Ckra18 | 0.093 | Ckra21 | Ckra29 | 0.298 | Ckra12 | Ckra21 | 0.587 | Ckra07 | Ckra27 | 0.869 |
| Ckra01 | Ckra12 | 0.095 | Ckra08 | Ckra24 | 0.301 | Ckra06 | Ckra12 | 0.588 | Ckra13 | Ckra29 | 0.876 |
| Ckra05 | Ckra27 | 0.095 | Ckra03 | Ckra18 | 0.308 | Ckra01 | Ckra13 | 0.591 | Ckra02 | Ckra03 | 0.888 |
| Ckra08 | Ckra13 | 0.100 | Ckra04 | Ckra18 | 0.313 | Ckra06 | Ckra18 | 0.593 | Ckra22 | Ckra29 | 0.892 |
| Ckra02 | Ckra29 | 0.102 | Ckra04 | Ckra29 | 0.316 | Ckra01 | Ckra05 | 0.601 | Ckra24 | Ckra29 | 0.897 |
| Ckra13 | Ckra22 | 0.109 | Ckra02 | Ckra07 | 0.317 | Ckra06 | Ckra21 | 0.612 | Ckra07 | Ckra21 | 0.909 |
| Ckra03 | Ckra06 | 0.112 | Ckra08 | Ckra29 | 0.318 | Ckra05 | Ckra13 | 0.625 | Ckra03 | Ckra22 | 0.932 |
| Ckra04 | Ckra21 | 0.120 | Ckra12 | Ckra22 | 0.325 | Ckra08 | Ckra12 | 0.647 | Ckra13 | Ckra21 | 0.939 |
| Ckra05 | Ckra08 | 0.126 | Ckra03 | Ckra27 | 0.354 | Ckra24 | Ckra27 | 0.647 | Ckra01 | Ckra18 | 0.945 |
| Ckra05 | Ckra21 | 0.142 | Ckra22 | Ckra24 | 0.355 | Ckra04 | Ckra08 | 0.660 | Ckra12 | Ckra13 | 0.946 |
| Ckra03 | Ckra05 | 0.150 | Ckra18 | Ckra27 | 0.381 | Ckra03 | Ckra12 | 0.666 | Ckra06 | Ckra13 | 0.968 |
| Ckra03 | Ckra08 | 0.160 | Ckra01 | Ckra22 | 0.396 | Ckra01 | Ckra24 | 0.681 | Ckra12 | Ckra29 | 0.971 |
| Ckra08 | Ckra21 | 0.166 | Ckra13 | Ckra27 | 0.417 | Ckra01 | Ckra08 | 0.682 | Ckra03 | Ckra07 | 0.974 |
| Ckra12 | Ckra24 | 0.169 | Ckra05 | Ckra29 | 0.441 | Ckra12 | Ckra18 | 0.686 | Ckra06 | Ckra29 | 0.974 |
