# Supplementary material for: Spatial population genetic structure of Caquetaia kraussii (Steindachner, 1878) evidenced by species-specific microsatellite loci in the middle and low basin of the Cauca River, Colombia
Source: PLoS One. 2024 Jun 4;19(6):e0304799. doi: 10.1371/journal.pone.0304799 (PMC11149877; doi:10.1371/journal.pone.0304799)
Supplement: S4 Table — (DOCX) [file pone.0304799.s006.docx]

|  | S8 | | | | | | S7 | | | | | | S6 | | | | | |
| --- | --- | --- | --- | --- | --- | --- | --- | --- | --- | --- | --- | --- | --- | --- | --- | --- | --- | --- |
| Locus | N | Na | Ho | He | P_HWE_ | FIS | N | Na | Ho | He | P_HWE_ | FIS | N | Na | Ho | He | P_HWE_ | FIS |
| Ckra01 | 26 | 21 | 0.962 | 0.959 | 0.160 | -0.003 | 49 | 23 | 1.000 | 0.952 | 0.629 | -0.051 | 80 | 24 | 0.975 | 0.944 | 0.895 | -0.033 |
| Ckra02 | 26 | 14 | 0.846 | 0.873 | 0.634 | 0.031 | 49 | 14 | 0.816 | 0.851 | 0.746 | 0.041 | 80 | 15 | 0.750 | 0.801 | **0.003** | 0.064 |
| Ckra03 | 26 | 13 | 0.923 | 0.855 | 0.909 | -0.081 | 49 | 13 | 0.837 | 0.878 | 0.334 | 0.047 | 80 | 12 | 0.950 | 0.878 | 0.063 | -0.083 |
| Ckra04 | 26 | 21 | 0.885 | 0.956 | 0.061 | 0.076 | 49 | 22 | 0.939 | 0.950 | 0.125 | 0.011 | 80 | 21 | 0.913 | 0.933 | 0.537 | 0.022 |
| Ckra05 | 26 | 17 | 0.923 | 0.941 | 0.507 | 0.020 | 49 | 17 | 0.918 | 0.921 | 0.139 | 0.003 | 80 | 18 | 0.975 | 0.922 | 0.475 | -0.058 |
| Ckra06 | 25 | 10 | 0.840 | 0.829 | 0.972 | -0.013 | 49 | 12 | 0.857 | 0.856 | 0.299 | -0.002 | 80 | 12 | 0.875 | 0.891 | 0.870 | 0.018 |
| Ckra07 | 25 | 19 | 0.880 | 0.949 | 0.281 | 0.074 | 49 | 22 | 0.939 | 0.946 | 0.530 | 0.008 | 80 | 20 | 0.938 | 0.937 | 0.315 | <**0.0001** |
| Ckra08 | 26 | 10 | 0.885 | 0.865 | 0.386 | -0.023 | 49 | 10 | 0.837 | 0.829 | 0.338 | -0.009 | 79 | 10 | 0.797 | 0.826 | 0.226 | 0.034 |
| Ckra12 | 26 | 10 | 0.923 | 0.877 | 0.969 | -0.054 | 49 | 10 | 0.898 | 0.853 | 0.890 | -0.053 | 80 | 10 | 0.800 | 0.844 | 0.112 | 0.052 |
| Ckra13 | 26 | 7 | 0.654 | 0.696 | 0.517 | 0.062 | 49 | 7 | 0.776 | 0.768 | 0.528 | -0.010 | 80 | 8 | 0.738 | 0.757 | 0.199 | 0.025 |
| Ckra18 | 26 | 11 | 0.962 | 0.876 | 0.475 | -0.099 | 49 | 13 | 0.857 | 0.873 | 0.220 | 0.018 | 79 | 13 | 0.861 | 0.846 | 0.358 | -0.018 |
| Ckra21 | 26 | 19 | 0.885 | 0.940 | 0.109 | 0.060 | 49 | 17 | 0.959 | 0.924 | 0.274 | -0.039 | 80 | 20 | 0.825 | 0.907 | 0.177 | 0.091 |
| Ckra22 | 26 | 16 | 0.885 | 0.918 | 0.754 | 0.037 | 49 | 15 | 0.878 | 0.908 | 0.298 | 0.034 | 80 | 16 | 0.850 | 0.893 | 0.671 | 0.049 |
| Ckra24 | 26 | 18 | 0.885 | 0.937 | 0.153 | 0.057 | 48 | 22 | 0.979 | 0.940 | 0.475 | -0.042 | 80 | 22 | 0.950 | 0.936 | 0.510 | -0.015 |
| Ckra27 | 26 | 8 | 0.731 | 0.811 | 0.703 | 0.101 | 48 | 9 | 0.792 | 0.777 | 0.714 | -0.020 | 80 | 9 | 0.738 | 0.764 | 0.533 | 0.035 |
| Ckra29 | 26 | 11 | 0.923 | 0.867 | 0.836 | -0.067 | 49 | 11 | 0.878 | 0.857 | 0.090 | -0.024 | 79 | 9 | 0.861 | 0.853 | 0.868 | -0.010 |

|  | S5 | | | | | | S4 | | | | | | PHI | | | | | |
| --- | --- | --- | --- | --- | --- | --- | --- | --- | --- | --- | --- | --- | --- | --- | --- | --- | --- | --- |
| Locus | N | Na | Ho | He | P_HWE_ | FIS | N | Na | Ho | He | P_HWE_ | FIS | N | Na | Ho | He | P_HWE_ | FIS |
| Ckra01 | 42 | 14 | 1.000 | 0.914 | 0.858 | -0.096 | 21 | 13 | 0.810 | 0.855 | 0.249 | 0.054 | 98 | 8 | 0.806 | 0.798 | 0.788 | -0.011 |
| Ckra02 | 43 | 9 | 0.744 | 0.780 | 0.327 | 0.046 | 21 | 8 | 0.714 | 0.751 | 0.436 | 0.051 | 100 | 6 | 0.700 | 0.671 | 0.073 | -0.044 |
| Ckra03 | 43 | 10 | 0.884 | 0.851 | 0.090 | -0.039 | 21 | 9 | 0.905 | 0.822 | 0.757 | -0.103 | 100 | 6 | 0.600 | 0.663 | 0.383 | 0.095 |
| Ckra04 | 42 | 17 | 0.810 | 0.885 | 0.622 | 0.087 | 21 | 13 | 0.762 | 0.900 | **0.008** | 0.157 | 99 | 10 | 0.717 | 0.656 | 0.248 | -0.094 |
| Ckra05 | 43 | 13 | 0.791 | 0.796 | 0.384 | 0.007 | 21 | 8 | 0.619 | 0.747 | 0.126 | 0.175 | 99 | 9 | 0.798 | 0.720 | 0.789 | -0.110 |
| Ckra06 | 43 | 8 | 0.744 | 0.788 | 0.203 | 0.057 | 21 | 8 | 0.810 | 0.724 | 0.476 | -0.122 | 100 | 7 | 0.470 | 0.436 | 0.825 | -0.079 |
| Ckra07 | 43 | 15 | 0.884 | 0.903 | 0.797 | 0.022 | 21 | 13 | 0.905 | 0.886 | 0.975 | -0.022 | 100 | 9 | 0.770 | 0.758 | 0.276 | -0.016 |
| Ckra08 | 43 | 8 | 0.884 | 0.801 | 0.132 | -0.105 | 21 | 7 | 0.524 | 0.732 | **0.001** | 0.289 | 100 | 6 | 0.690 | 0.721 | 0.482 | 0.044 |
| Ckra12 | 43 | 8 | 0.860 | 0.809 | 0.492 | -0.065 | 21 | 6 | 0.762 | 0.779 | 0.370 | 0.023 | 100 | 5 | 0.690 | 0.654 | 0.983 | -0.055 |
| Ckra13 | 43 | 7 | 0.791 | 0.697 | 0.958 | -0.136 | 21 | 6 | 0.619 | 0.567 | 0.663 | -0.095 | 100 | 4 | 0.540 | 0.560 | 0.070 | 0.036 |
| Ckra18 | 43 | 11 | 0.791 | 0.774 | 0.376 | -0.022 | 21 | 7 | 0.667 | 0.650 | 0.873 | -0.026 | 100 | 7 | 0.800 | 0.784 | 0.521 | -0.020 |
| Ckra21 | 42 | 15 | 0.929 | 0.889 | 0.337 | -0.045 | 19 | 16 | 0.789 | 0.925 | 0.152 | 0.150 | 100 | 7 | 0.670 | 0.706 | 0.603 | 0.051 |
| Ckra22 | 43 | 12 | 0.837 | 0.865 | **0.009** | 0.032 | 21 | 9 | 0.714 | 0.787 | 0.117 | 0.095 | 99 | 8 | 0.747 | 0.795 | 0.408 | 0.060 |
| Ckra24 | 43 | 19 | 0.907 | 0.917 | 0.364 | 0.011 | 21 | 17 | 0.810 | 0.940 | **0.003** | 0.141 | 100 | 12 | 0.740 | 0.789 | **0.004** | 0.062 |
| Ckra27 | 43 | 6 | 0.651 | 0.632 | 0.069 | -0.031 | 21 | 6 | 0.429 | 0.568 | 0.094 | 0.250 | 99 | 6 | 0.434 | 0.415 | 0.977 | -0.047 |
| Ckra29 | 43 | 10 | 0.860 | 0.870 | 0.585 | 0.011 | 20 | 9 | 0.800 | 0.832 | 0.426 | 0.039 | 99 | 6 | 0.444 | 0.554 | 0.134 | 0.198 |

|  | Stock S6-S7-S8 | | | | | | Stock S4-S5 | | | | | |
| --- | --- | --- | --- | --- | --- | --- | --- | --- | --- | --- | --- | --- |
| Locus | N | Na | Ho | He | P_HWE_ | FIS | N | Na | Ho | He | P_HWE_ | FIS |
| Ckra01 | 155 | 28 | 0.981 | 0.949 | 0.916 | -0.034 | 63 | 16 | 0.937 | 0.901 | 0.605 | -0.039 |
| Ckra02 | 155 | 17 | 0.787 | 0.830 | 0.116 | 0.052 | 64 | 10 | 0.734 | 0.767 | 0.241 | 0.042 |
| Ckra03 | 155 | 15 | 0.910 | 0.881 | 0.326 | -0.033 | 64 | 11 | 0.891 | 0.844 | 0.265 | -0.055 |
| Ckra04 | 155 | 24 | 0.916 | 0.945 | **0.048** | 0.030 | 63 | 18 | 0.794 | 0.902 | **0.013** | 0.121 |
| Ckra05 | 155 | 19 | 0.948 | 0.926 | 0.760 | -0.025 | 64 | 13 | 0.734 | 0.778 | 0.155 | 0.056 |
| Ckra06 | 154 | 13 | 0.864 | 0.880 | 0.800 | 0.019 | 64 | 8 | 0.766 | 0.766 | 0.560 | <**0.0001** |
| Ckra07 | 154 | 24 | 0.929 | 0.941 | 0.080 | 0.014 | 64 | 17 | 0.891 | 0.897 | 0.983 | 0.007 |
| Ckra08 | 154 | 11 | 0.825 | 0.834 | 0.304 | 0.011 | 64 | 9 | 0.766 | 0.795 | **0.033** | 0.037 |
| Ckra12 | 155 | 12 | 0.852 | 0.852 | 0.224 | <**0.0001** | 64 | 8 | 0.828 | 0.798 | 0.339 | -0.039 |
| Ckra13 | 155 | 9 | 0.735 | 0.753 | **0.014** | 0.023 | 64 | 7 | 0.734 | 0.656 | 0.921 | -0.120 |
| Ckra18 | 154 | 15 | 0.877 | 0.864 | 0.106 | -0.015 | 64 | 11 | 0.750 | 0.738 | 0.197 | -0.017 |
| Ckra21 | 155 | 22 | 0.877 | 0.921 | 0.374 | 0.048 | 61 | 19 | 0.885 | 0.905 | 0.695 | 0.022 |
| Ckra22 | 155 | 19 | 0.865 | 0.905 | 0.611 | 0.045 | 64 | 12 | 0.797 | 0.846 | **0.017** | 0.059 |
| Ckra24 | 154 | 25 | 0.948 | 0.941 | 0.194 | -0.008 | 64 | 21 | 0.875 | 0.924 | 0.123 | 0.053 |
| Ckra27 | 154 | 9 | 0.753 | 0.784 | 0.571 | 0.039 | 64 | 8 | 0.578 | 0.610 | 0.063 | 0.053 |
| Ckra29 | 154 | 12 | 0.877 | 0.856 | 0.608 | -0.024 | 63 | 11 | 0.841 | 0.862 | 0.363 | 0.024 |
