# Supplementary material for: Spatial population genetic structure of Caquetaia kraussii (Steindachner, 1878) evidenced by species-specific microsatellite loci in the middle and low basin of the Cauca River, Colombia
Source: PLoS One. 2024 Jun 4;19(6):e0304799. doi: 10.1371/journal.pone.0304799 (PMC11149877; doi:10.1371/journal.pone.0304799)
Supplement: S5 Table — PO: posterior probability. Values in bold denote statistical significance. (DOCX) [file pone.0304799.s007.docx]

| Locus | PHI/S4-S5 | PHI/S6-S7-S8 | S4-S5/S6-S7-S8 |
| --- | --- | --- | --- |
| Ckra01 | 0.036 | 0.033 | 0.047 |
| Ckra02 | 0.051 | 0.051 | 0.043 |
| Ckra03 | 0.045 | 0.049 | 0.041 |
| Ckra04 | 0.040 | 0.047 | 0.304 |
| Ckra05 | 0.054 | 0.048 | 0.053 |
| Ckra06 | 0.069 | 0.046 | 0.101 |
| Ckra07 | 0.121 | 0.052 | 0.237 |
| Ckra08 | 0.050 | 0.042 | 0.047 |
| Ckra12 | 0.069 | 0.045 | 0.047 |
| Ckra13 | 0.054 | 0.064 | 0.059 |
| Ckra18 | 0.107 | 0.055 | 0.049 |
| Ckra21 | 0.053 | 0.038 | **0.972** |
| Ckra22 | 0.043 | 0.048 | 0.042 |
| Ckra24 | 0.225 | 0.214 | 0.228 |
| Ckra27 | 0.096 | 0.045 | 0.113 |
| Ckra29 | 0.085 | 0.063 | 0.182 |
