# Supplementary material for: Spatial population genetic structure of Caquetaia kraussii (Steindachner, 1878) evidenced by species-specific microsatellite loci in the middle and low basin of the Cauca River, Colombia
Source: PLoS One. 2024 Jun 4;19(6):e0304799. doi: 10.1371/journal.pone.0304799 (PMC11149877; doi:10.1371/journal.pone.0304799)
Supplement: S6 Table — PC: Paired comparisons, rxy: Relatedness. (DOCX) [file pone.0304799.s008.docx]

| **Relatedness** |  | **Unrelated samples**  **(0<rxy<0.125)** | **Full sibs**  **(0.125< rxy< 0.4375)** | **Half sibs /** **Parent–offspring**  **(rxy> 0.4375)** | **Total** |
| --- | --- | --- | --- | --- | --- |
| **Global** | CP | 69642 | 10111 | 1250 | 81003 |
|  | % | 85.975 | 12.482 | 1.543 |  |
| **PHI** | CP | 416 | 4073 | 461 | 4950 |
|  | % | 8.404 | 82.283 | 9.313 |  |
| **S4** | CP | 140 | 69 | 1 | 210 |
|  | % | 66.667 | 32.857 | 0.476 |  |
| **S5** | CP | 698 | 205 | 0 | 903 |
|  | % | 77.298 | 22.702 | 0.000 |  |
| **S6** | CP | 2661 | 495 | 4 | 3160 |
|  | % | 84.209 | 15.665 | 0.127 |  |
| **S7** | CP | 1040 | 136 | 0 | 1176 |
|  | % | 88.435 | 11.565 | 0.000 |  |
| **S8** | CP | 298 | 27 | 0 | 325 |
|  | % | 91.692 | 8.308 | 0.000 |  |
| **Stock S4-S5** | CP | 1533 | 482 | 1 | 2016 |
|  | % | 76.042 | 23.909 | 0.050 |  |
| **Stock S6-S7-S8** | CP | 10547 | 1384 | 4 | 11935 |
|  | % | 88.370 | 11.596 | 0.034 |  |
