# Supplementary material for: Spatial population genetic structure of Caquetaia kraussii (Steindachner, 1878) evidenced by species-specific microsatellite loci in the middle and low basin of the Cauca River, Colombia
Source: PLoS One. 2024 Jun 4;19(6):e0304799. doi: 10.1371/journal.pone.0304799 (PMC11149877; doi:10.1371/journal.pone.0304799)
Supplement: S7 Table — (DOCX) [file pone.0304799.s009.docx]

| **Comparison** | **Migration rate** | **Standard Deviation** | **Migration Direction** |
| --- | --- | --- | --- |
| m[S4][S5] | 0.258 | 0.027 | Upstream |
| m[S5][S6] | 0.018 | 0.014 | Upstream |
| m[S4][S6] | 0.016 | 0.015 | Upstream |
| m[S4][S7] | 0.012 | 0.012 | Upstream |
| m[S4][S8] | 0.012 | 0.012 | Upstream |
| m[S5][S7] | 0.007 | 0.007 | Upstream |
| m[S5][S8] | 0.007 | 0.007 | Upstream |
| m[S6][S7] | 0.006 | 0.005 | Upstream |
| m[S7][S8] | 0.006 | 0.006 | Upstream |
| m[PHI][S5] | 0.004 | 0.003 | Upstream |
| m[S6][S8] | 0.004 | 0.004 | Upstream |
| m[PHI][S4] | 0.003 | 0.003 | Upstream |
| m[PHI][S6] | 0.003 | 0.003 | Upstream |
| m[PHI][S7] | 0.003 | 0.003 | Upstream |
| m[PHI][S8] | 0.003 | 0.003 | Upstream |
| m[PHI][PHI] | 0.981 | 0.008 | Same |
| m[S6][S6] | 0.963 | 0.013 | Same |
| m[S5][S5] | 0.940 | 0.021 | Same |
| m[S4][S4] | 0.679 | 0.012 | Same |
| m[S8][S8] | 0.677 | 0.010 | Same |
| m[S7][S7] | 0.673 | 0.006 | Same |
| m[S7][S6] | 0.295 | 0.015 | Downstream |
| m[S8][S6] | 0.262 | 0.025 | Downstream |
| m[S8][S5] | 0.019 | 0.015 | Downstream |
| m[S6][S5] | 0.015 | 0.010 | Downstream |
| m[S4][PHI] | 0.012 | 0.012 | Downstream |
| m[S8][S7] | 0.012 | 0.012 | Downstream |
| m[S8][PHI] | 0.010 | 0.010 | Downstream |
| m[S8][S4] | 0.010 | 0.010 | Downstream |
| m[S5][PHI] | 0.008 | 0.008 | Downstream |
| m[S7][S5] | 0.008 | 0.008 | Downstream |
| m[S5][S4] | 0.007 | 0.007 | Downstream |
| m[S7][PHI] | 0.006 | 0.006 | Downstream |
| m[S7][S4] | 0.006 | 0.006 | Downstream |
| m[S6][PHI] | 0.005 | 0.004 | Downstream |
| m[S6][S4] | 0.004 | 0.004 | Downstream |
