# Supplementary material for: Spatial population genetic structure of Caquetaia kraussii (Steindachner, 1878) evidenced by species-specific microsatellite loci in the middle and low basin of the Cauca River, Colombia
Source: PLoS One. 2024 Jun 4;19(6):e0304799. doi: 10.1371/journal.pone.0304799 (PMC11149877; doi:10.1371/journal.pone.0304799)
Supplement: Attachment — Submitted filename: Response to reviewers.docx [file pone.0304799.s010.docx]

Response to reviewers

Dear Editor,

We really appreciate the detailed revision of the reviewers and have edited the manuscript following your valuable recommendations, which have led to improve our paper.

We hope that the manuscript is now suitable for publication in Plos One.

PONE-D-23-37840

Spatial population genetic structure of Caquetaia kraussii (Steindachner, 1878) evidenced by species-specific microsatellite loci in the middle and low basin of the Cauca River, Colombia

PLOS ONE

Dear Dr. Marquez,

Thank you for submitting your manuscript to PLOS ONE. After careful consideration, we feel that it has merit but does not fully meet PLOS ONE’s publication criteria as it currently stands. Therefore, we invite you to submit a revised version of the manuscript that addresses the points raised during the review process.

Both reviewer's have some valid critisizm of the analyses and the manuscript that need to be addressed before acceptance. Please revise the manuscript accordingly and carefully consider the comments regarding the soundness of the analyses.

Please submit your revised manuscript by Mar 08 2024 11:59PM. If you will need more time than this to complete your revisions, please reply to this message or contact the journal office at plosone@plos.org. When you're ready to submit your revision, log on to https://www.editorialmanager.com/pone/ and select the 'Submissions Needing Revision' folder to locate your manuscript file.

Please include the following items when submitting your revised manuscript:

A rebuttal letter that responds to each point raised by the academic editor and reviewer(s). You should upload this letter as a separate file labeled 'Response to Reviewers'.

A marked-up copy of your manuscript that highlights changes made to the original version. You should upload this as a separate file labeled 'Revised Manuscript with Track Changes'.

An unmarked version of your revised paper without tracked changes. You should upload this as a separate file labeled 'Manuscript'.

If you would like to make changes to your financial disclosure, please include your updated statement in your cover letter. Guidelines for resubmitting your figure files are available below the reviewer comments at the end of this letter.

Please see below.

If applicable, we recommend that you deposit your laboratory protocols in protocols.io to enhance the reproducibility of your results. Protocols.io assigns your protocol its own identifier (DOI) so that it can be cited independently in the future. For instructions see: https://journals.plos.org/plosone/s/submission-guidelines#loc-laboratory-protocols. Additionally, PLOS ONE offers an option for publishing peer-reviewed Lab Protocol articles, which describe protocols hosted on protocols.io. Read more information on sharing protocols at https://plos.org/protocols?utm_medium=editorial-email&utm_source=authorletters&utm_campaign=protocols.

Complete information is documented in the manuscript.

We look forward to receiving your revised manuscript.

Kind regards,

Sven Winter

Academic Editor

PLOS ONE

Journal Requirements:

When submitting your revision, we need you to address these additional requirements.

1. Please ensure that your manuscript meets PLOS ONE's style requirements, including those for file naming. The PLOS ONE style templates can be found at

https://journals.plos.org/plosone/s/file?id=wjVg/PLOSOne_formatting_sample_main_body.pdf and

https://journals.plos.org/plosone/s/file?id=ba62/PLOSOne_formatting_sample_title_authors_affiliations.pdf

2. In your Methods section, please provide additional information regarding the permits you obtained for the work. Please ensure you have included the full name of the authority that approved the field site access and, if no permits were required, a brief statement explaining why.

Done. Now: “This study assessed fin and muscle tissues preserved in ethanol 95%, obtained from 403 *C. kraussii* individuals captured in different sectors of the Cauca River (**Figure 1**) by Universidad de Antioquia, Universidad de Córdoba, and Universidad Nacional de Colombia Sede Medellín, through scientific cooperation agreement CT-2019-000661, under environmental license # 0155 of January 30th, 2009, from Ministry of Environment, Housing and Territorial Development for the Ituango hydroelectric construction.”

3. Note from Emily Chenette, Editor in Chief of PLOS ONE, and Iain Hrynaszkiewicz, Director of Open Research Solutions at PLOS: Did you know that depositing data in a repository is associated with up to a 25% citation advantage (https://doi.org/10.1371/journal.pone.0230416)? If you’ve not already done so, consider depositing your raw data in a repository to ensure your work is read, appreciated and cited by the largest possible audience. You’ll also earn an Accessible Data icon on your published paper if you deposit your data in any participating repository (https://plos.org/open-science/open-data/#accessible-data).

Done. Now we include “S2 Table. Genotype data at 16 microsatellite loci included in the population genetic analysis of *Caquetaia kraussi*. First row indicates respectively: Number of loci, Number of individuals, Number of sampling sections, Sample size fo seven sections. Third row indicates respectively: Sample ID, Sampling section, Genetic stock, Locus name for 16 loci.”

4. Thank you for stating the following in the Acknowledgments Section of your manuscript:

"This study was funded by Universidad Nacional de Colombia Sede Medellín and Empresas Públicas de Medellín, Grant CT-2019-000661 “Variabilidad genética de un banco de peces de los sectores medio y bajo del Río Cauca”."

We note that you have provided funding information that is not currently declared in your Funding Statement. However, funding information should not appear in the Acknowledgments section or other areas of your manuscript. We will only publish funding information present in the Funding Statement section of the online submission form.

Please remove any funding-related text from the manuscript and let us know how you would like to update your Funding Statement. Currently, your Funding Statement reads as follows:

"This study was funded by Universidad Nacional de Colombia Sede Medellín and Empresas Públicas de Medellín, Grant CT-2019-000661 “Variabilidad genética de un banco de peces de los sectores medio y bajo del Río Cauca”. Funders have not play any role in the study design, data collection and analysis, decision to publish, or preparation of the manuscript."

Please include your amended statements within your cover letter; we will change the online submission form on your behalf.

Done. We have removed the text, and we would like to retain the current Funding Statement.

5. Please include your full ethics statement in the ‘Methods’ section of your manuscript file. In your statement, please include the full name of the IRB or ethics committee who approved or waived your study, as well as whether or not you obtained informed written or verbal consent. If consent was waived for your study, please include this information in your statement as well.

Please see response above.

6. We note that [Figure 1] in your submission contain [map/satellite] images which may be copyrighted. All PLOS content is published under the Creative Commons Attribution License (CC BY 4.0), which means that the manuscript, images, and Supporting Information files will be freely available online, and any third party is permitted to access, download, copy, distribute, and use these materials in any way, even commercially, with proper attribution. For these reasons, we cannot publish previously copyrighted maps or satellite images created using proprietary data, such as Google software (Google Maps, Street View, and Earth). For more information, see our copyright guidelines: http://journals.plos.org/plosone/s/licenses-and-copyright.

We require you to either (1) present written permission from the copyright holder to publish these figures specifically under the CC BY 4.0 license, or (2) remove the figures from your submission:

a. You may seek permission from the original copyright holder of Figure 1 to publish the content specifically under the CC BY 4.0 license.

We recommend that you contact the original copyright holder with the Content Permission Form (http://journals.plos.org/plosone/s/file?id=7c09/content-permission-form.pdf) and the following text:

“I request permission for the open-access journal PLOS ONE to publish XXX under the Creative Commons Attribution License (CCAL) CC BY 4.0 (http://creativecommons.org/licenses/by/4.0/). Please be aware that this license allows unrestricted use and distribution, even commercially, by third parties. Please reply and provide explicit written permission to publish XXX under a CC BY license and complete the attached form.”

Please upload the completed Content Permission Form or other proof of granted permissions as an "Other" file with your submission.

In the figure caption of the copyrighted figure, please include the following text: “Reprinted from [ref] under a CC BY license, with permission from [name of publisher], original copyright [original copyright year].”

b. If you are unable to obtain permission from the original copyright holder to publish these figures under the CC BY 4.0 license or if the copyright holder’s requirements are incompatible with the CC BY 4.0 license, please either i) remove the figure or ii) supply a replacement figure that complies with the CC BY 4.0 license. Please check copyright information on all replacement figures and update the figure caption with source information. If applicable, please specify in the figure caption text when a figure is similar but not identical to the original image and is therefore for illustrative purposes only.

The following resources for replacing copyrighted map figures may be helpful:

USGS National Map Viewer (public domain): http://viewer.nationalmap.gov/viewer/

The Gateway to Astronaut Photography of Earth (public domain): http://eol.jsc.nasa.gov/sseop/clickmap/

Maps at the CIA (public domain): https://www.cia.gov/library/publications/the-world-factbook/index.html and https://www.cia.gov/library/publications/cia-maps-publications/index.html

NASA Earth Observatory (public domain): http://earthobservatory.nasa.gov/

Landsat: http://landsat.visibleearth.nasa.gov/

USGS EROS (Earth Resources Observatory and Science (EROS) Center) (public domain): http://eros.usgs.gov/#

Natural Earth (public domain): http://www.naturalearthdata.com/

Now, we provide a new figure 1. “**Figure 1**. *Caquetaia kraussii* sampled sites in the Cauca River, Colombia.

PHI: Ituango hydroelectric project, 17 sites (purple). ECrPSC1: Santa Cruz fish farming station, 1 site (gray). S4: 3 sites (light blue). S5: 5 sites (blue). S6: 13 sites (red). S7: 3 sites (orange). S8: 2 sites (yellow). Self-made creation of the map based on contour lines scaled 1:100,000 from the Instituto Geográfico Agustín Codazzi source, 2019 (Available from: IGAC Geoportal, https://geoportal.igac.gov.co/contenido/datos-abiertos-cartografia-y-geografia).”

7. Please include captions for your Supporting Information files at the end of your manuscript, and update any in-text citations to match accordingly. Please see our Supporting Information guidelines for more information: http://journals.plos.org/plosone/s/supporting-information.

Done. Legends of the Supporting Information files are now at the end of the manuscript.

[Note: HTML markup is below. Please do not edit.]

Reviewers' comments:

Reviewer's Responses to Questions

Comments to the Author

1. Is the manuscript technically sound, and do the data support the conclusions?

The manuscript must describe a technically sound piece of scientific research with data that supports the conclusions. Experiments must have been conducted rigorously, with appropriate controls, replication, and sample sizes. The conclusions must be drawn appropriately based on the data presented.

Reviewer #1: Partly

Reviewer #2: Yes

2. Has the statistical analysis been performed appropriately and rigorously?

Reviewer #1: No

Reviewer #2: Yes

3. Have the authors made all data underlying the findings in their manuscript fully available?

The PLOS Data policy requires authors to make all data underlying the findings described in their manuscript fully available without restriction, with rare exception (please refer to the Data Availability Statement in the manuscript PDF file). The data should be provided as part of the manuscript or its supporting information, or deposited to a public repository. For example, in addition to summary statistics, the data points behind means, medians and variance measures should be available. If there are restrictions on publicly sharing data—e.g. participant privacy or use of data from a third party—those must be specified.

Reviewer #1: No

Reviewer #2: Yes

4. Is the manuscript presented in an intelligible fashion and written in standard English?

PLOS ONE does not copyedit accepted manuscripts, so the language in submitted articles must be clear, correct, and unambiguous. Any typographical or grammatical errors should be corrected at revision, so please note any specific errors here.

Reviewer #1: Yes

Reviewer #2: Yes

5. Review Comments to the Author

Please use the space provided to explain your answers to the questions above. You may also include additional comments for the author, including concerns about dual publication, research ethics, or publication ethics. (Please upload your review as an attachment if it exceeds 20,000 characters)

Reviewer #1: Comments to: “Spatial population genetic structure of Caquetaia kraussii (Steindachner, 1878) evidenced by species-specific microsatellite loci in the middle and low basin of the Cauca River, Colombia” by IC Tenorio, CD Joya and EJ Márquez.

This MS describe population genetic structure and genetic diversity of a cichlid species. Using variability of 16 microsatellite loci the authors found high genetic diversity and four genetic clusters.

The MS is well written and easy to follow. I have comments.

1.- The manuscript is not in the format of the journal. Authors should consider the submission instructions for their papers.

2.- MS lacks page number and line numbers

Done

Introduction

3.- Page 1 introduction. Please add a reference to: “Moreover, individuals that migrate and reproduce with distant populations exhibit gene flow and, thus, high genetic diversity.”

Done. Now: “Moreover, individuals that migrate and reproduce with distant populations exhibit gene flow and, thus, high genetic diversity [24,25].”

4.- “Particularly, this study aimed to provide a response to C. kraussii genetic diversity and

structure related questions between 2020 and 2022”. I cannot find where the authors used date (2020-2022) in the analysis. Please explain or delete.

Done. Now: “Namely, this study aimed to provide a response to *C. kraussii* genetic diversity and structure related questions in the medium and lower sections of the Cauca River”

Materials and methods

5.- “Furthermore, a discriminant analysis of principal components (DAPC) was

performed using the R-package Adegenet (Jombart, 2008), with six principal components

retained.” How the authors selected 6 PCA? How many DL were used? The authors used de a-score?

This idea is incomplete. Now: “Furthermore, a discriminant analysis of principal components (DAPC) was performed using the R-package Adegenet (Jombart, 2008), with 32 principal components and six discriminant functions retained.”

a-score: 0.422.

6.- “Other approach included a Bayesian analysis in STRUCTURE 2.3.4 (Pritchard et al., 2000), with 1,000,000 Monte Carlo Markov chains with 100.000 regarded as the burn-in period.” The authors used the admixture model? The authors used correlated or uncorrelated allele frequencies?

Done. Now: “Other approach included a Bayesian analysis in STRUCTURE 2.3.4 [94], with 1,000,000 Monte Carlo Markov chains with 100,000 regarded as the burn-in period, admixture model and correlated allele frequencies as a priori models.”

7.- “BayeScan v2.1 (Foll & Gaggiotti, 2008) was used for loci detection under selection.” BayeScan could be used before all analysis.

BayeScan was run prior the other analysis. We reordered its position in the methodology. For clarity, we also rewrite a level 2 heading: “Microsatellites development and detection of outlier loci”

8.- “Lastly, GENECLASS2 (Piry et al., 2004) was used for testing whether an individual resides in the sampled site or is immigrant from another section. To this end, L_home/L_max rate was used for first generation migrant detection.” Why you expect first generation migrant? Please use other methods (e.g. BayesAss).

We made a mistake. We deleted “To this end, L_home/L_max rate was used for first generation migrant detection.”

Also,

In Materials and methods: “Likewise, the BA3MSAT extension of the BAYESASS software [111,112] was employed for the recent estimation of gene flow between sections, utilizing 50,000,000 MCMC, with a burn-in of 5,000,000 and sampling intervals of 5,000. Delta values for migration rates (deltaM), allele frequencies (deltaA), and inbreeding coefficients (deltaF) were set at 0.49, 0.39, and 0.46, respectively. Convergence was assessed using the Tracer v1.7.2 program [113].”

In Results: “Results from BayesAss (**S6 Table**) showed that the highest migration rates were observed within each section, ranging from 67.3% to 98.1% (m[PHI][PHI]: 0.981±0.008, m[S6][S6]: 0.963±0.013, m[EPSC][EPSC]: 0.967±0.011, m[S5][S5]: 0.940±0.021, m[S4][S4]: 0.679±0.012, m[S8][S8]: 0.677±0.010, m[S7][S7]: 0.673±0.006). Additionally, a higher migration rate upstream (25.8%) was observed between S4 and S5, while higher migration rates downstream (26.2% - 29.5%) were observed between S6, S7, and S8. The remaining migration rates ranged from 0.3% to 1.9%.”

In Discussion: “The low dispersion potential of the species and its larvae because of parental care, the variation in its dispersion behavior (upstream in the S4-S5 stock and downstream in the S6-S7-S8 stock), and the confluence magnitude of the Nechí River and the Cauca River may explain the existence of two *C. kraussii* stocks, one upstream and other downstream of the Nechí River mouth”

Results:

9.- “Genetic diversity showed a decreasing gradient from the lower to the middle section of the Cauca River (S8>S7>S6>S5>S4)” Please add a figure for this result.

Done. We include the reference of the Table 2 and S1 Figure: “Genetic diversity showed a decreasing gradient from the lower to the middle section of the Cauca River (S8>S7>S6>S5>S4; **Table 2; S1 Figure**)”

10.- “for both He (R: -0.970, p: 0.001) and Ar (R: 1.000, p: 0.000)”. Please, never a p-value = 0. Probability in this case could be p < 0.0001.

Done

11.- Table 2.: please careful with p-value. Change from 0.0000 to <0.0001.

Done

12.- Table 2: Change from “PHWE: statistical significance tests of departure from Hardy-Weinberg equilibrium.” To PHWE: p-value for the departure from Hardy-Weinberg equilibrium. Same for FIS p-value

Done

13.- “Furthermore, Bayescan posterior probability (PO) values for the loci detection under selection (Supplementary Table 4) evidenced that the only paired comparison showing one locus under selection (Ckra 21; reference value > 0.76) was S4-S5 v. S6-S7-S8. Moreover, another parameter found, Alpha, had a value of 1.234 which suggests the existence of balancing or purifying selection. It is important in the analysis this detection? Do you erase these loci? No words in discussion about that?

Now, we included: “The results presented below are based on 16 loci, as consistent findings were obtained when including or excluding this locus in subsequent analyses.”

14.- “(Supplementary Figure 2)”: I cannot find this figure.

This figure is renamed as “S2_Fig.tif”

Discussion

Please re-write this section considering a whole discussion follow the main goal of the study. Discussion about each method used cannot be useful for readers. For example, main results are about population genetic structure. Thus, what about the respective role of the barriers and fish behavior, etc.

Done. We reordered the paragraphs and removed the subheadings.

Discussion about Ne is poor. Please improve this section comparing with other species.

Now: “Studies on effective population sizes in cichlids are relatively limited. Within the examined area, certain low-migration-range species (*Astyanax caucanus*, [131]) and medium-migration-range species (*Prochilodus magdalenae*, [120]) exhibit high effective population sizes. However, *Pseudoplatytoma magdaleniatum*, also classified as a medium-migration-range species, displayed effective population sizes below 1000 [49].”

Conclusion

Please re-write considering main results.

Now: “This study unveils a spatial structure within *C. kraussii*, comprising four genetic groups that exhibit high genetic diversity compared to neotropical cichlids. Both natural stocks, located upstream and downstream the Nechí River mouth, display high effective population size, indicating a high long-term evolutionary potential. Nevertheless, the dam and fish farm groups, probably originated by founder effect, are susceptible to potential harmful effects because of their low effective population size and high relatedness degree. Additionally,…”

References

Please change “Referencias” to “References”

Done

Change format to plosone

Done

Figures

Change quality (300 dpi)

Done

Reviewer #2: The manuscript contains an interesting study on the genetic diversity and populations structure of the cichlid Caquetaia Krausii located in the basin of the Causa River. The topic is worthy of investigation, and the manuscript is particularly interesting in that it investigates the effect of Ituango hydroelectric project on the genetic structure of the species.

In principle, the experimental design is appropriate, except that the study of EPSC stock does not fit in. The objective of the manuscript is to determine the genetic diversity of the species and the genetic structure of the population. In my opinion, the main factor in examining the genetic structure is the impact of the hydroelectric project on the natural populations, and the farm stock does not correspond to the natural stock.

Due to the lack of previous information of the population genetics of this species, the farm stock constitutes a reference for all parameters measured in the population genetic analyses.

The structure of the introduction is appropriate; however, I suggest including a paragraph on the hydroelectric project and the effect of such projects on the genetic diversity of cichlids.

Done. Now: “The impact of hydroelectric plants on cichlids in neotropical environments is poorly documented and fish responses appear to depend on their life history [35], the duration of fragmentation [31], and potential impacts resulting from hybridization between invasive or introduced species [34]. For example, *Gymnogeophagus setequedas*, an endangered cichlid that seems to prefer fast waters, was reported to have disappeared after the construction of the Itaipu reservoir [51]. However, it exhibited gene flow in lotic environments of the Iguaçu River basin [35]. For populations fragmented for approximately 17 years or longer, *Geophagus* aff. *brasiliensis* and other neotropical non-migratory fish species show significant genetic structure [31]. Recent evidence of hybridization between two introduced species in a reservoir (*Cichla ocellaris* var. *kelberi* and *Cichla piquiti*) and the high genetic diversity found using microsatellite loci raises concerns about its indication of a possible increase in local adaptability that could enhance establishment success in novel areas [34].”

The description of the methodology needs some corrections:

Please add sample sizes in the materials and methods. They could be found only in Table 2 in the results.

Done. Now: “These sections, previously identified [65] include the medium (PHI: 100 samples) and lower (S4, S5, S6, S7 and S8: 21, 43, 80, 49 and 26 samples, respectively) sections of the Cauca River.”

The 16 microsatellite markers were selected from 30 “according to their polymorphism level” – this means that 14 were monomorphic, or that the PIC value was not enough high?

Now: “A total of 16 (4mer: 15; 5mer: 1) out of 30 preselected loci satisfied the polymorphic criteria (**Table 1**). The remaining loci either exhibited inconsistent amplification, low levels of polymorphism, or were monomorphic (**S1 Table**).”

Please add the description of the Mantel test to the materials and methods.

“Finally, two Mantel tests were performed using the Ecodist package in the R program [99] to examine the spatial correlation of genetic distances (F’_st_ and Jost's D_EST_ estimators) within the evaluated sections. Geographical distances were calculated based on the nearest geographical coordinates between sections, excluding EPSC as it does not correspond to a natural group.”

Please add the proportion of SMM in TPM, the variance of TPM and the number of iterations for the Wilcoxon signed rank test.

Now: “…and two-phase (TPM; parameter settings: IAM: 10%, SMM: 90%, Variance: 10.00, Probability: 90%) models, through Wilcoxon signed rank test with 1000 iterations [101]…”

Results:

The authors give a negative correlation between Ar and geographical distance, but the value of R is 1.000 (based on the figure, this does not seem realistic). Please revise it.

Now: “…and Ar (R: -0.980, p < 0.0001).”

In Table 2, are the values means of the 16 markers? If yes, then please include SD also. Was there a statistical comparison between populations? If yes, significant differences should be marked and taken into account in the diversity gradient in the text.

Done. Please see the edited Table 2.

The resolution of Figure 2 is low, making the figure difficult to interpret.

Done. Please see the new Figure 2.

Additionally to the first-generation migrant detection, I suggest calculating the relative directional migration network by the method based on Jost's D using divMigrate-online software (Sundqvist et al., 2016). In addition to a statistical assessment of one-way migration, this also provides a good and easy-to-understand chart to illustrate the extent of migration.

We performed the BayesAss analysis as indicated by the one of the reviewers (please see above). Although divMigrate-online software (Sundqvist et al., 2016) is easily implemented, the genetic data of *C. kraussii* did not satisfy to the idealized conditions of DivMigrate (Sundqvist et al., 2016) since the population sizes were not equal. Furthermore, the implementation of DivMigrate is challenging in complex scenarios such as recent founder effects or common ancestry and populations that are not in equilibrium.

I recommend running the mantel test without the farm stock, as it is expected to improve the correlation. Moreover, it might be also useful to calculate only the stocks under the hydroelectric project separately, taking into account its strong impact.

This test did not include the farm stock. For clarity, a new paragraph was included in the methodology: “Finally, two Mantel tests were performed using the Ecodist package in the R program (Goslee & Urban, 2007) to examine the spatial correlation of genetic distances (F’_st_ and Jost's D_EST_ estimators) within the evaluated sector. Geographical distances were calculated based on the nearest geographical coordinates between sectors, excluding EPSC as it does not correspond to a natural group.”

Discussions should focus less on the reproductive features of the species and more on the habitat characteristics that may cause differences in population size and diversity of subpopulations. This is particularly valid for the hydroelectric project (founder effect, relatedness, migration rate, etc.).

Now: “Spatial structure due to events such as the interruption of migratory pathways are not expected for sedentary fish species, as they typically exhibit a spatial genetic structure associated with their life history. In such cases, the potential threats associated with hydroelectric constructions may be related to factors that affect fish species downstream, such as fluctuations in nutrient transportation and shifts in oxygen concentrations [115], water quality and temperature, sediment accumulation, variations in water flow, alterations in the morphology of the main river channel and swamps [116–119]. The impact of these factors on the population genetics of *C. kraussi* remains unknown due to the lack of previous information on these subjects. However, the significant gene flow observed in various migratory fish species coexisting in this area [48–50,120–122] suggests the absence of physical or chemical barriers limiting gene flow in these species.”.

Nevertheless, after the necessary corrections, the manuscript could provide valuable results for the population genetics of Cichlid fishes.

6. PLOS authors have the option to publish the peer review history of their article (what does this mean?). If published, this will include your full peer review and any attached files.

If you choose “no”, your identity will remain anonymous but your review may still be made public.

Do you want your identity to be public for this peer review? For information about this choice, including consent withdrawal, please see our Privacy Policy.

Reviewer #1: No

Reviewer #2: No

[NOTE: If reviewer comments were submitted as an attachment file, they will be attached to this email and accessible via the submission site. Please log into your account, locate the manuscript record, and check for the action link "View Attachments". If this link does not appear, there are no attachment files.]

While revising your submission, please upload your figure files to the Preflight Analysis and Conversion Engine (PACE) digital diagnostic tool, https://pacev2.apexcovantage.com/. PACE helps ensure that figures meet PLOS requirements. To use PACE, you must first register as a user. Registration is free. Then, login and navigate to the UPLOAD tab, where you will find detailed instructions on how to use the tool. If you encounter any issues or have any questions when using PACE, please email PLOS at figures@plos.org. Please note that Supporting Information files do not need this step.
